# Time-Frequency Distribution Map-Based Convolutional Neural Network (CNN) Model for Underwater Pipeline Leakage Detection Using Acoustic Signals

**DOI:** 10.3390/s20185040

**Published:** 2020-09-04

**Authors:** Yingchun Xie, Yucheng Xiao, Xuyan Liu, Guijie Liu, Weixiong Jiang, Jin Qin

**Affiliations:** College of Engineering, Ocean University of China, Qingdao 266000, China; xycharbour@163.com (Y.X.); liuxuyan@stu.ouc.edu.cn (X.L.); liuguijie@ouc.edu.cn (G.L.); brilliant25@163.com (W.J.); oucqinjin@163.com (J.Q.)

**Keywords:** acoustic leak signal, hydrophone, fault diagnosis, time-frequency image, EEMD, CNN

## Abstract

Detection technology of underwater pipeline leakage plays an important role in the subsea production system. In this paper, a new method based on the acoustic leak signal collected by a hydrophone is proposed to detect pipeline leakage in the subsea production system. Through the pipeline leakage test, it is found that the radiation noise is a continuous spectrum of the medium and high-frequency noise. Both the increase in pipe pressure and the diameter of the leak hole will narrow the spectral structure and shift the spectrum center towards the low frequencies. Under the same condition, the pipe pressure has a greater impact on the noise; every 0.05 MPa increase in the pressure, the radiation sound pressure level increases by 6-7 dB. The time-frequency images were obtained by processing the acoustic signals using the Ensemble Empirical Mode Decomposition (EEMD) and Hilbert–Huang transform (HHT), and fed into a two-layer Convolutional Neural Network (CNN) for leakage detection. The results show that CNN can correctly identify the degree of pipeline leakage. Hence, the proposed method provides a new approach for the detection of pipeline leakage in underwater engineering applications.

## 1. Introduction

The subsea production system is a technology-intensive field of marine engineering. It is one of the leading technologies in modern marine petroleum engineering [1,2]. Subsea production systems commonly use high pressure to secure the delivery of oil and gas, and the equipment faces the deformation and destruction in high temperature and high-pressure environments. The transportation of oil and gas also contains the acid corrosive gas and a large number of impurities. Corrosive gases (such as H_2_S, CO_2_, etc.) can lead to pipeline corrosion and failure, while the impurities can cause erosion and wear on the surface of the pipes, leading to pipeline leaks [3]. Pipeline erosion and corrosive wear are the main causes of submarine oil and gas pipeline failure.

Currently, the pipeline leakage detection often adopts the ship-borne underwater robots [4]. The advantages of manpower inspection are comprehensive and accurate, but there are also some disadvantages. On the one hand, inspections cannot monitor pipeline leakage in real-time. On the other hand, one inspection operation not only needs a large number of professional technicians and equipment but also needs a long duration of time, high cost, and favorable weather.

Most detection techniques rely on the measurement of a physical quantity or reaction to a physical phenomenon, such as the acoustic, flow rate, pressure, gas sampling, optics, etc., and their combinations [5,6,7,8,9]. Among the current fault diagnosis methods for the oil and gas pipeline leakage, some are only suitable for the specific operating environment (e.g., soil–gas sampling method [10]) or mainly designed for gas leakage (e.g., ultrasonic flowmeter [11]); moreover, some sensors are difficult to install and the maintenance is costly (e.g., distributed optical fiber sensor method [12] and cable sensor [13]). Methods for infusion pipeline design (e.g., digital signal processing method [14]) need to retrofit existing pipelines. Especially, the fluid-model-based method [15,16] requires modeling of the piping system, and is suitable for small leakage and location. Because the acoustic wave propagates farthest as a mechanical wave in the ocean, underwater sound carries the noise information of the pipeline leakage and can be used to monitor the leakage in real-time.

In recent years, the hydrophone is widely used in the obstacle avoidance of underwater vehicles [17], marine monitoring [18,19], oil quality diagnosis [20], marine seismic exploration [21] and underwater target positioning [22], etc. Piskur et al. [17] designed a kind of underwater acoustic system based on the hydrophone sensor for biomimetic underwater vehicles to avoid collision, and only two hydrophones were used to successfully avoid obstacles. The sensitivity of the hydrophone and the number of hydrophones in the array play important roles. The hydrophone is an important part of the underwater acoustic signal acquisition (UASA), and the UASA node is widely used in underwater noise monitoring, antisubmarine, and observation of marine animals [18]. Hydrophone can convert the acoustic signal into the electrical signal, which is easy to process by existing signal processing systems. The underwater signal includes plenty of characteristics. In a maritime environmental management system, Enguix et al. [19] used the intelligent hydrophone to record the underwater impulse noise to study the underwater noise pollution and its influence on marine biology.

With the development of computers, the competence of computing has been enhanced greatly. The methods of combining fault information with machine learning algorithms have attracted the attention of scholars. Kumar et al. [23] designed a method of symmetric single-valued neutrosophic cross-entropy (SVNCE) of Mode Decomposition (VMD) to classify the bearing defects in the centrifugal pump. They also used the analytical wavelet transform (AWT) to obtain grayscale acoustic images, and then fed them into an improved CNN to create a fault diagnosis model [24]. Both methods produced high recognition accuracy. In the underwater, Luna et al. [20] proposed a new method to diagnose the quality of mineral oil, and they used a homemade hydrophone to get signals from sound waves traveling through different kinds of oil. Then, they created a dataset for these oil samples and used a supervised machine learning technique to categorize oil samples. Shen et al. [25] used the hydrophone to capture the radiated noise produced by ships, and design a CNN with several auditory-like mechanisms to classify the ship type. The recognition accuracy was 87.2%. They proposed the construction of a deep network to improve the auditory inspired CNN; thus, the trained auditory CNN model has sensitive auditory neurons to identify specific spectral-temporal patterns in sound.

The pipeline leakage radiation noise signal has the characteristics of nonlinearity and nonstationarity. The traditional method usually processes the signal from the frequency domain or the time-domain, and cannot effectively extract the signal leakage features. The time-frequency analysis method is a combination of time and frequency domains and can accurately describe the local characteristics of nonlinear and nonstationary signals [26]. In this aspect, classical Hilbert–Huang Transform (HHT) [27] and Ensemble Empirical Mode Decomposition (EEMD) [28] algorithms are widely used. However, HHT and EEMD have not been combined with deep learning to detect underwater pipeline leakage. To address this issue, a fault diagnosis method based on the acoustic leak signal is proposed in this paper. The contributions of this work are as follows.

1. The structure of the jet generated by the underwater pipeline leakage is studied.
2. The influence of the pipe pressure and leakage hole size on the radiated noise is analyzed.
3. Radiated noise signals are demonstrated in the time-frequency domain through the EEMD algorithm, and the pipeline leakage degree is identified by HHT-CNN.

## 2. Simulation of the Underwater Pipeline Leak

In the gas pipeline leakage, the noise generated by the jet field can be regarded as a quadrupole sound source [29,30]. In 1952, Lighthill [31,32] proposed an acoustic analogy method based on the gas motion equation. As an indirect method, this method combined the governing equation of the fluid motion (Navier–Stokes equation) into a wave equation with a source and is the first theoretical model used to predict the jet noise.

Lighthill acoustic analogy explains the relationship between the acoustic wave propagation and fluid parameters. The acoustic radiation equation for the fluid motion is

(1) $$\left(\frac{\partial^{2}}{\partial t^{2}}-c_{0}{}^{2}\nabla^{2}\right)\rho =\frac{\partial^{2}T_{ij}}{\partial x_{i}\partial x_{j}}$$

where $T_{ij}$ is the stress tensor, namely the radiation of the quadrupole sound source, $x_{i}$ and $x_{j}$, respectively, are the physical spaces. According to Lighthill’s eighth power velocity theory, the total sound power is proportional to the eighth power jet velocity and is proportional to the square of the jet diameter, and the power of the jet radiated noise is

(2) $$W=\frac{KD^{2}\rho_{0}U^{8}}{c_{0}^{5}}$$

where *K* is the Lighthill constant, *D* is the diameter of the leak hole, $\rho_{0}$ is the density, *U* is the leakage velocity, and $c_{0}$ is the speed of sound. The sound power *W* and the sound intensity *I* and the envelope area of the sound source *S* satisfy the following equation.

(3) W=IS

The sound intensity *I* and the maximum sound pressure p of the spherical waves in the sound field satisfy the following equation.

(4) $$I=\frac{p^{2}}{\rho_{0}c_{0}}$$

The quadrupole sound source propagates as a spherical wave, with $S=\pi D^{2}$. By coupling Equations (2)–(4), the maximum sound pressure can be derived as follows:

(5) $$p=\sqrt{\frac{K}{\pi }}\frac{\rho_{0}U^{4}}{c_{0}{}^{2}}$$

According to Lighthill acoustic analogy, the sound source of the underwater pipeline leakage is mainly determined by the flow field density and particle velocity. When the pipeline of the underwater production system leaks, the fluid in the pipe is ejected from the leakage hole under the action of the huge pressure difference, and the sound source of the jet is dominant in the radiated noise caused by leakage. Jet noise sources consist of the following three main components.

1. The high-velocity jet fluid is injected into static or relatively low-speed fluid media. The two-fluid media are rapidly mixed due to a large velocity difference. Therefore, turbulent pulsation will produce strong jet noise in the boundary layer.
2. Due to the high velocity of injection, the jet flow will generate many vortices to form turbulence. Thus, the jet fluid will produce strong turbulence noise, which eventually becomes the radiation noise source.
3. In the vicinity of the leakage port, the turbulent noise is generated due to the existence of a high-velocity gradient region.

**Figure 1:**
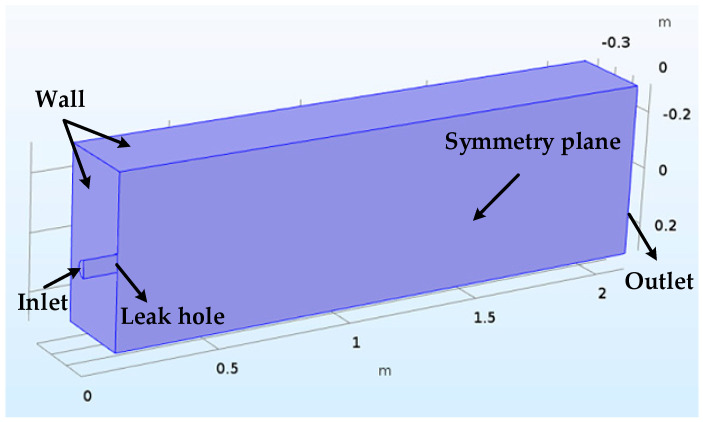
The flow field model.

There has been a lot of simulation and experimental studies on the prediction of pneumatic jet noise [30]. In this paper, the CFD simulation software COMSOL is used to establish a 2000 mm × 600 mm × 600 mm rectangular flow field. The RNG k-ε turbulence model is adopted. The leak hole is circular and set at the center of the cross-section. The diameters are 20, 60, 100, and 140 mm, respectively. The inlet boundary condition of the flow field uses velocity inlet with 10, 20, 30 and 40 m/s, respectively. The outlet boundary condition is a pressure outlet, and the other boundary conditions of the surfaces are nonsliding wall surfaces. Because the flow field is uniformly axis-symmetric, to improve the calculation efficiency, the flow field is cut along the XOZ plane, and one half is taken for the simulation analysis. The cutting surface is set as symmetric boundary conditions, as shown in Figure 1.

**Figure 2:**
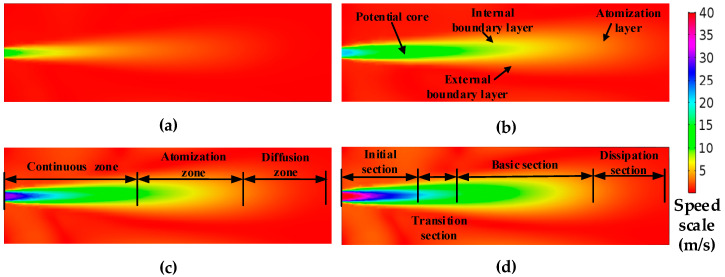
Steady-state flow field velocity cloud map with different jet velocities (leakage hole 60 mm). (**a**) 10 m/s; (**b**) 20 m/s; (**c**) 30m/s; (**d**) 40 m/s.

**Figure 3:**
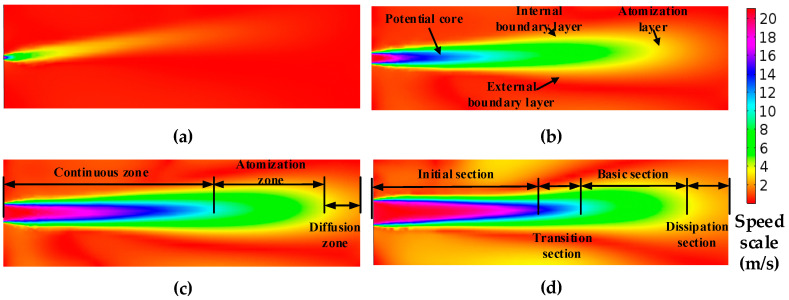
Steady-state flow field velocity cloud map with different leakage hole (at 0.3 Mpa). (**a**) 20 m; (**b**) 60 m; (**c**) 100 m; (**d**) 140 m.

When the number of the iterations reaches a certain degree, the difference between inlet flow and outlet flow is 1.7%, and the iteration convergence is considered. Underwater jet is the process of converting the pressure energy into the jet flow energy, different jet pressure, and different leakage aperture velocity variation cloud maps are shown in Figure 2 and Figure 3, where the structural distribution of the flow field is clearly observed.

**Figure 4:**
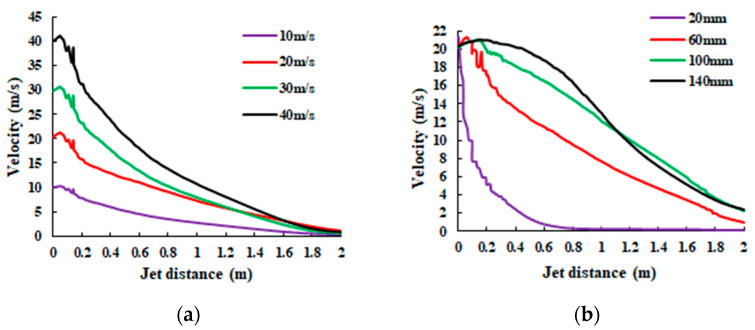
Velocity change on the centerline of the flow field: (**a**) different inlet velocity; (**b**) different leak hole size.

The variations of the jet centerline velocity with different inlet velocities and different leakage holes are shown in Figure 4.

By analyzing the results of Figure 2, Figure 3 and Figure 4 in combination with the Lighthill acoustic analogy, it can be concluded that:

1. The flow field structure of the underwater jet can be divided into three flow zones: Continuous flow zone: this zone is located from the jet nozzle to five diameters away from the jet nozzle. The center is the core of the jet, and the core is intensely mixed with the static fluid with high turbulence intensity and high-frequency radiation noise. Atomization flow zone: this zone is from the end of the core of the jet to the radius 10 times from the jet hole. The turbulence intensity and flow velocity decrease with the increase of distance, also the frequency of radiation noise decreases. Diffusion flow zone: following the atomization flow zone, the turbulence intensity and flow velocity continue to decrease.
2. From Figure 2 and Figure 3, the acoustic radiation comes mainly from the core of the jet, and the high-velocity fluid mixes with the absorbed fluid to form a highly turbulent and directional fluid. In the continuous flow area, the velocity gradient from the core to the mixing boundary is large, and there are complex and changeable stresses in the fluid, with high turbulence intensity, and the flow velocity and pressure of the fluid vary rapidly, thus producing strong radiation noise.
3. In Figure 4, the velocity of the fluid in the direction of the jet decreases gradually as the volume of the fluid increases, but a small amount of high-speed fluid is retained at the jet outlet, and its velocity still keeps the outlet velocity of the leak outlet, which becomes the core of the jet. The instantaneous velocity of the leak at the same pressure increases with the leak diameter.

## 3. Acoustics Image Acquisition

**Figure 5:**
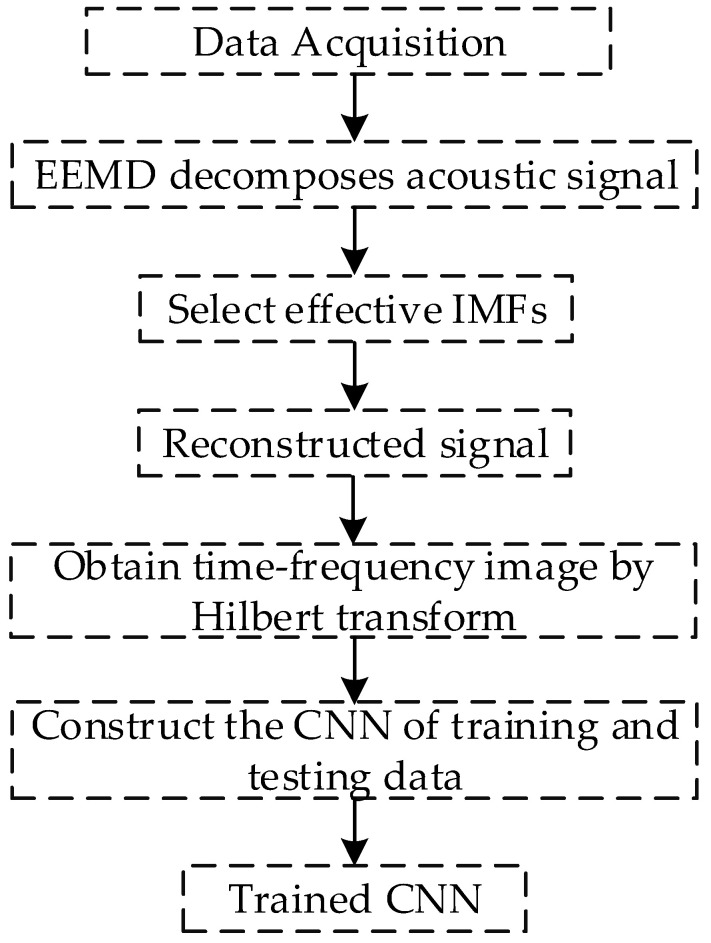
Process diagram.

An overview of the proposed method for underwater pipeline leakage detection is shown in Figure 5. The process begins with the acquisition of the acoustic signal by a hydrophone, and the time-frequency image connects the acoustics with CNN.

The EEMD algorithm [28] can be expressed as

(6) $$x_{0}\left(t\right)=\sum_{i=1}^{m}{\mathrm{IMF}}_{i}\left(t\right)+res\left(t\right)$$

where $x_{0}(t)$ is the original signal, ${\mathrm{IMF}}_{i}(t)$ is the i-th Intrinsic Mode Function (IMF) component of the original signal, and res(t) is the residue of the original signal.

After EEMD decomposition, the correlation analysis is conducted between the IMF and original signal, and the denoised signal is obtained after reconstruction. The correlation coefficients between each IMF component and the original signal are obtained according to Equation (7).

(7) $$\rho_{xy}=\frac{\sum_{i=1}^{N}\left(x_{i}-\bar{x}\right)\left(y_{i}-\bar{y}\right)}{\sqrt{\sum_{i=1}^{N}{\left(x_{i}-\bar{x}\right)}^{2}}\sqrt{\sum_{i=1}^{N}{\left(y_{i}-\bar{y}\right)}^{2}}}$$

When the correlation coefficient is obtained, filter the sensitive IMF components according to the threshold calculation Equation (8) [33].

(8) $$u_{h}=\frac{\max\left(u_{i}\right)}{10\times \max\left(u_{i}\right)-3}$$

where $u_{h}$ is the threshold and $u_{i}$ is the correlation coefficient between the *i*-th IMF component and the original signal. Take $u_{i}>u_{h}$ (IMF_i_) as an effective IMF_k_ to reconstruct the signal as

(9) $$x_{re}(t)=\sum_{k=1}^{k}{\mathrm{IMF}}_{k}(t)$$

Acoustic image (Hilbert spectrum) are obtained by HHT [27] as

(10) $$\hat{x_{re}}(t)=H[x_{re}(t)]=\frac{1}{\pi }\int_{-\infty }^{+\infty }\frac{x_{re}(\tau )}{t-\tau }d\tau$$

## 4. The Underwater Experiment of Radiation Noise

The experimental field is a straight-wall reinforced concrete structure, with a length and width 12 m × 6 m, the maximum working water depth of 1.5 m. The applied apparatus is shown in Table 1.

**Figure 6:**
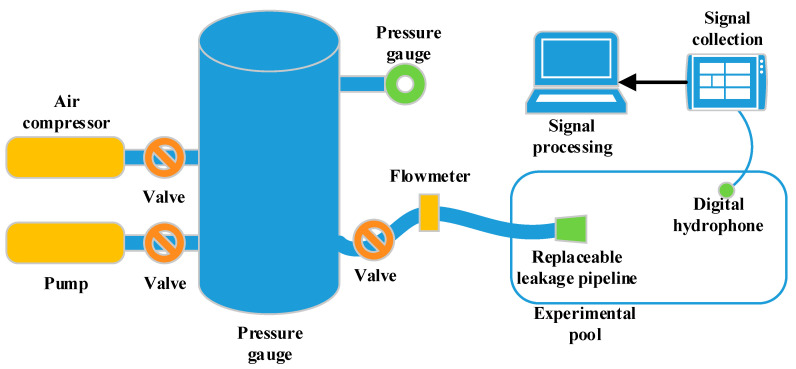
Equipment diagram.

**Figure 7:**
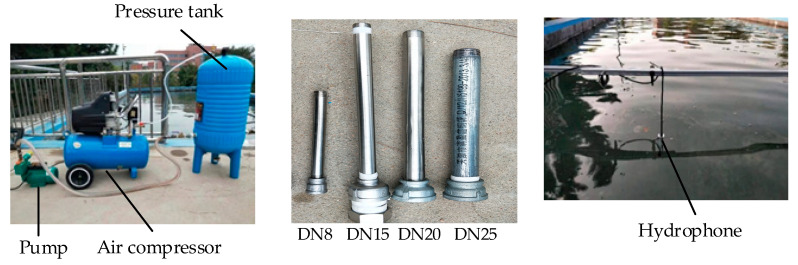
Test on site.

In the present test, a self-priming pump was used to add liquid to the pressure tank, then the compressed gas was added to the pressure tank. The air compressor and valve were adjusted to control the pressure in the pressure tank to create jets at different speeds. The sampling frequency of the hydrophone was set at 48 kHz. The test device connection is shown in Figure 6. The field device assembly is shown in Figure 7.

The experiment used leakage pressure and leakage caliber as variables, and the pressure in the pressure tank varied from 0.15 to 0.30 MPa. The nominal diameters of replaceable leak pipelines are shown in Table 2.

**Figure 8:**
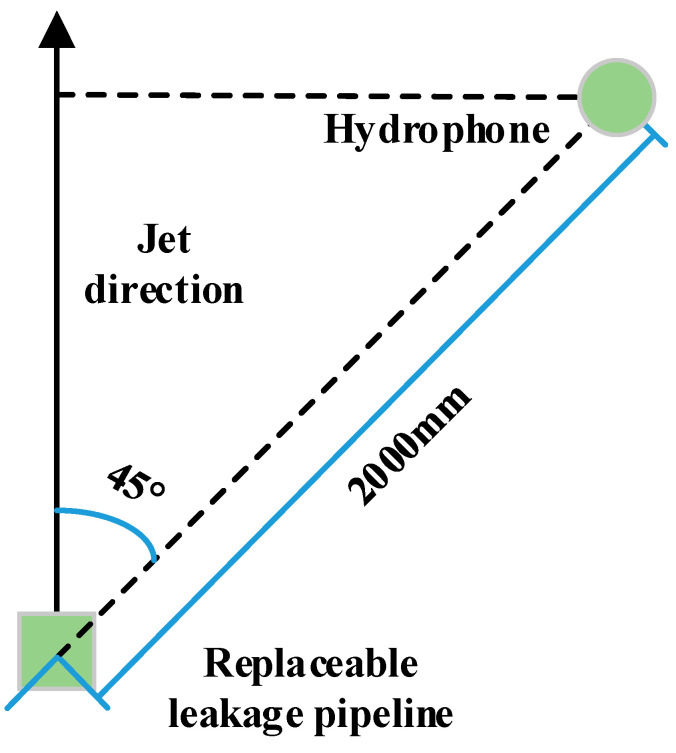
Hydrophone arrangement.

As is shown in Figure 8, when a hydrophone is arranged, the digital hydrophone and the jet direction should be misaligned, which prevents the hydrophone from being too close to the jet flow and forming pseudosound due to the impact of the water flow. For the jet radiation noise test, a leak hole was fixed in the center of the test pool, 0.6 m away from the water surface. The digital hydrophone was 2 m away from the jet hole and the direction of the jet was 45°.

## 5. Analysis and Results

### 5.1. Signal Decomposition

**Figure 9:**
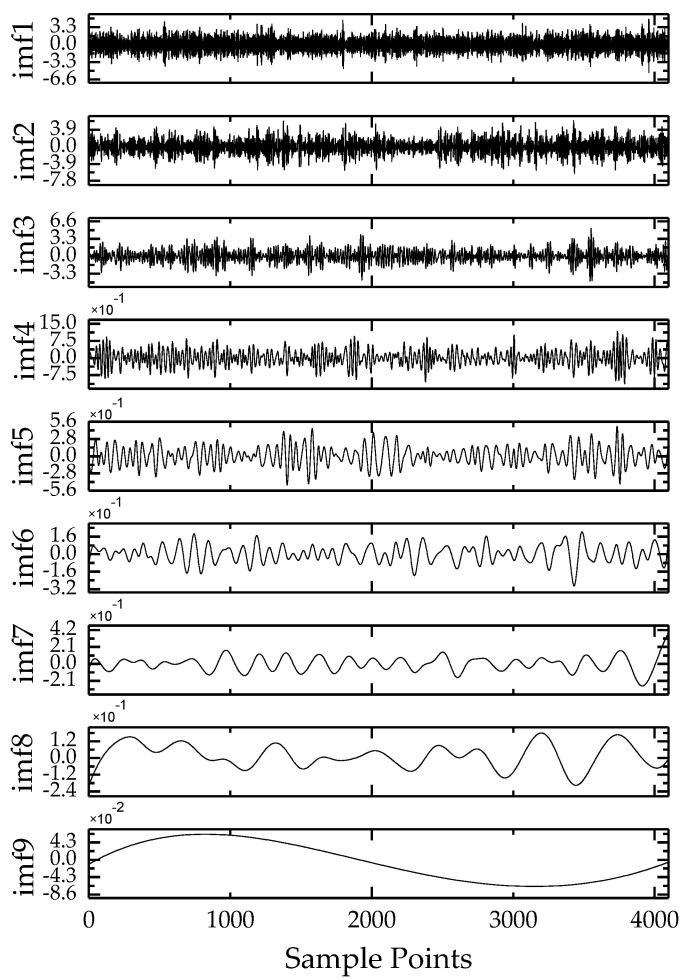
EEMD decomposition results of underwater acoustic signals.

EEMD was used to decompose the leaked sound radiation signal. The DN20 pipe leaked at a pressure of 0.25 MPa was taken as an example. There are nine IMF components, as shown in Figure 9.

### 5.2. Filtering and Refactoring

**Figure 10:**
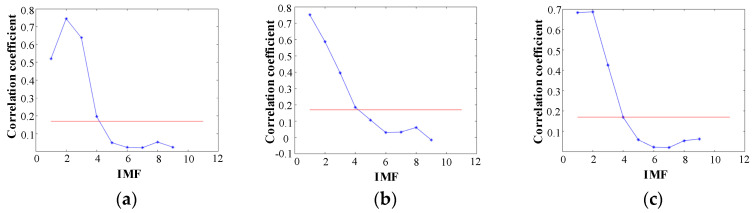

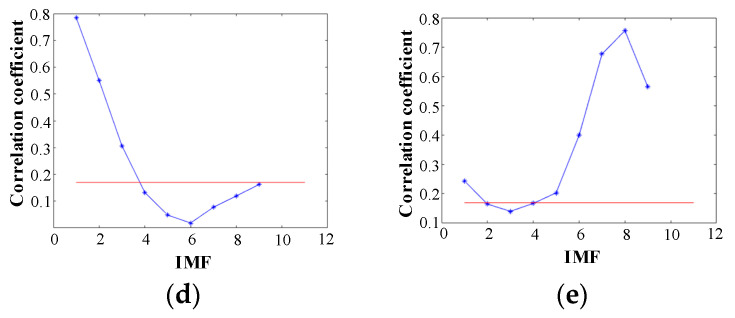
Correlation coefficient and threshold of each Intrinsic Mode Function (IMF) component in DN20. (**a**) 0.30 MPa; (**b**) 0.25 MPa; (**c**) 0.20 MPa; (**d**) 0.15 MPa; (**e**) Not leak.

The effective IMFs were chosen according to Equations (7) and (8). As shown in Figure 10, the acoustic signals with different leakage pressure conditions of the DN20 pipe were selected as an example to compare with the nonleakage signals. The red line in the figure was drawn according to the threshold value. When the pipeline leaks, the main contribution of the noise is the first three or four IMF components of the middle and high frequencies. Without leakage, the correlation coefficients of the second to fourth IMF components are extremely low.

**Figure 11:**
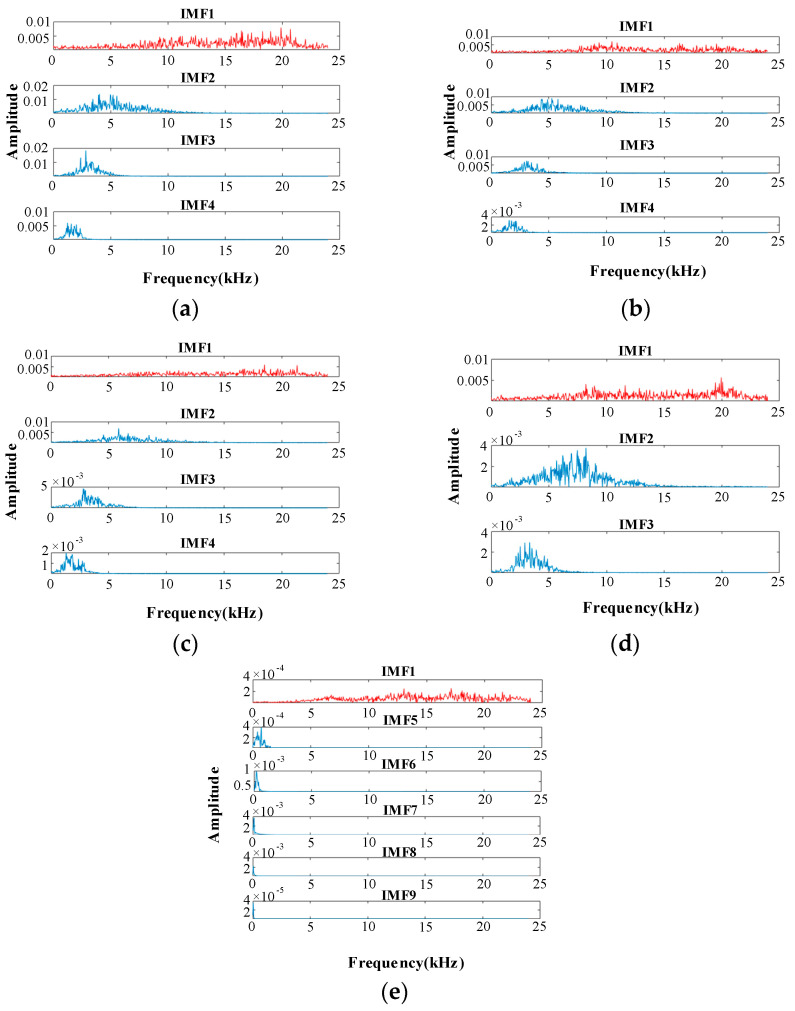
The various effective IMF component spectra of DN20. (**a**) 0.30 MPa; (**b**) 0.25 MPa; (**c**) 0.20 MPa; (**d**) 0.15 MPa; (**e**) Not leak.

The IMF components above the threshold value are selected as the effective IMF components, and the frequency domain analysis is performed on these IMF components. For example, the spectrum in Figure 11d corresponds to the first three IMFs in Figure 10d. The spectrum contains abundant information about the leakage.

**Figure 12:**
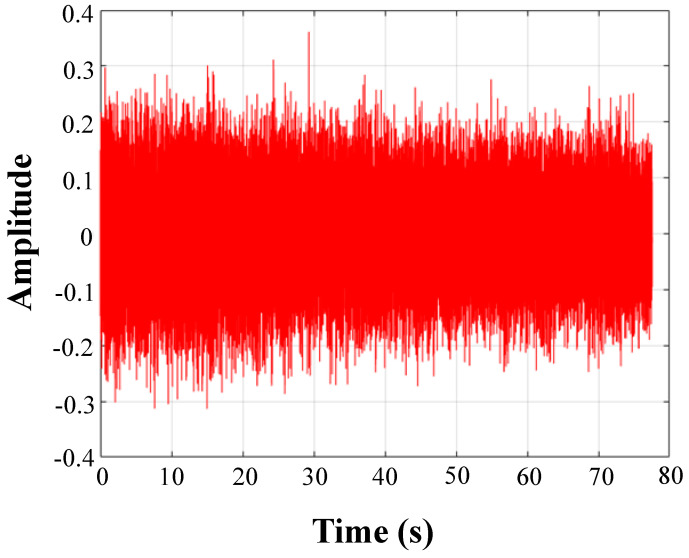
The original signal.

**Figure 13:**
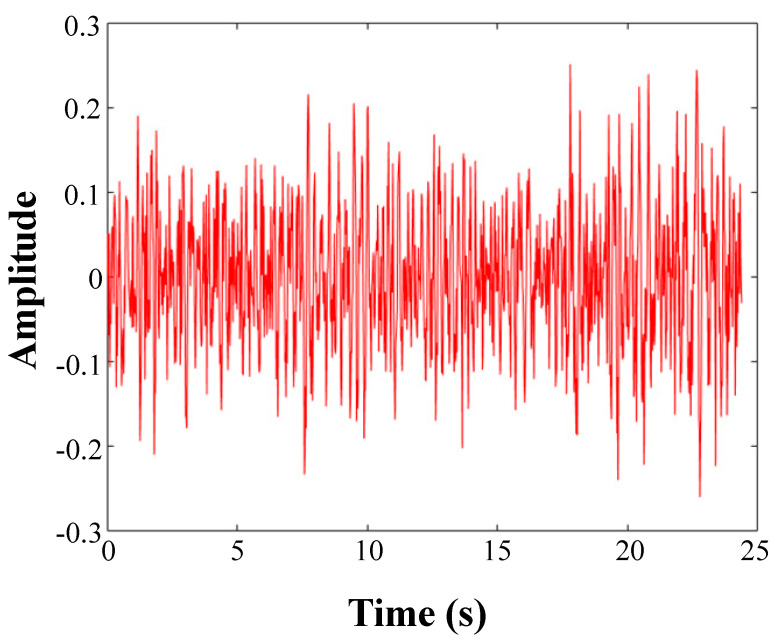
Partial signal reconstruction.

In Figure 11, IMF1 is red and the rest of the IMF is blue; IMF1 includes the full spectrum information. The IMF1 component of each signal includes high-frequency noise components above 13 kHz. Moreover, there is also a high-frequency component of the IMF1 component in the nonleaked signal. Therefore, a low-pass filter is used to eliminate the influence of the high-frequency noise components above 13 kHz in the IMF1 component. The signal is then reconstructed with effective IMF components to obtain the underwater pipeline leak acoustic radiation reconstruction signal. The waveform in time-domain is shown in Figure 12 and Figure 13 (e.g., DN20 at 0.25 MPa). The device will generate additional noise when it first starts up, which is also reflected in the early stage in Figure 12.

### 5.3. Time-Frequency Map

**Figure 14:**
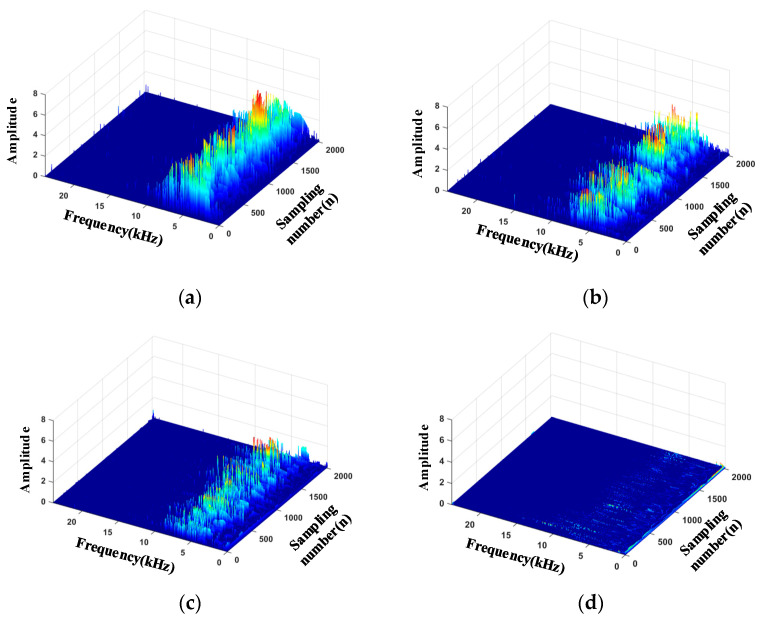
Three-dimensional (3D) time-frequency diagram of each leak state in DN20. (**a**) 0.30 MPa; (**b**) 0.25 MPa; (**c**) 0.20 MPa; (**d**) Not leak

Hilbert transform was performed on the reconstructed signal to make a time-frequency map to describe the variation of the signal amplitude with time and frequency over the entire frequency range. The energy distribution of the radiated acoustic signals in different leakage states on the Hilbert map is different, as shown in Figure 14. One can see that under the same leak hole, with the increase of pressure, the energy increases. At 0.20 MPa, energy is mainly distributed in the 0–10 kHz band, especially around 6 kHz. Compared with 0.20 MPa, the energy distribution frequency band is narrower at 0.25 MPa, and the center frequency has a downward trend. At 0.30 MPa, the energy is obviously concentrated around 5 kHz. These differences create the possibility of identification of the leakages.

### 5.4. Results and Analysis of Underwater Pipeline Leakage Noise

#### 5.4.1. Effect of Different Leakage Pressures on the Sound Pressure Level of Jet Radiation

**Figure 15:**
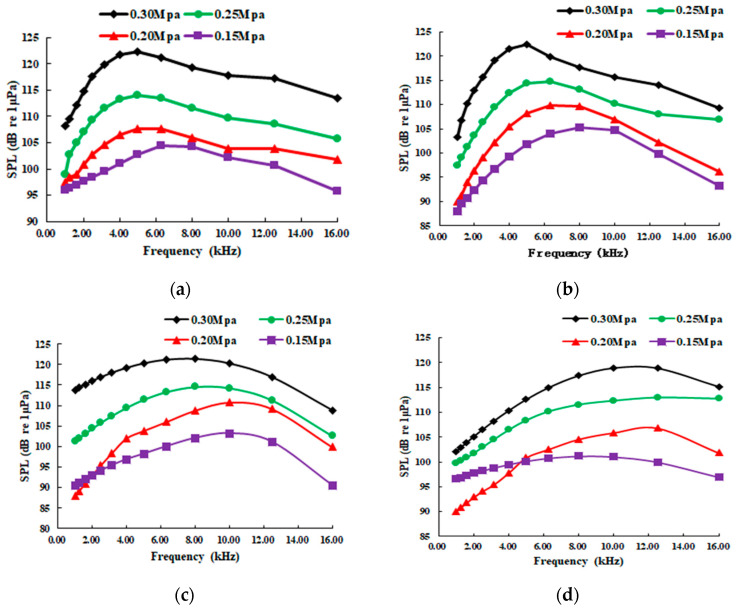
Radiated sound pressure levels under different leakage pressure. (**a**) DN25; (**b**) DN20; (**c**) DN15; (**d**) DN8.

A-weighted 1/3 octave processing and Gaussian quadratic fitting were performed on the denoising sound pressure signal. Figure 15 shows the sound pressure level (SPL) of different leakage pressures with a 45° jet direction and 2 m distance.

The main conclusions from Figure 15 are as follows: (1) From the pipe leakage radiation noise test, the sound signal caused by the leakage is a broadband signal. The radiated noise generated by the pipeline leakage is a continuous spectrum noise in the middle and high frequencies. The center of the spectrum is related to the pressure of the leakage pipeline. In the present work, the center frequency is between 5 and 13 kHz. (2) As the pressure of the leakage pipeline increases, the amplitude of the radiated noise SPL also increases at the same location in the far-field. For every 0.05 MPa increase in pressure, the radiated sound pressure level increases by 6-7 dB. (3) As the pressure increases, a frequency bandwidth of the radiated noise becomes narrower, and its center frequency moves toward the low-value.

#### 5.4.2. Effect of Different Leakage Diameters on Jet Radiation Sound Pressure Level

**Figure 16:**
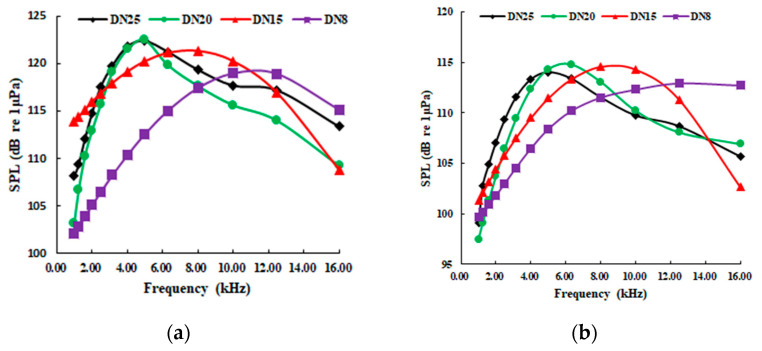

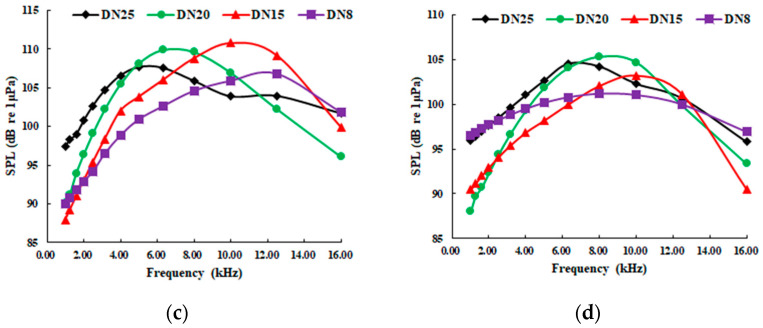
Radiation sound pressure levels with different leak diameters. (**a**) 0.30 MPa; (**b**) 0.25 MPa; (**c**) 0.20 MPa; (**d**) 0.15MPa.

Figure 16 shows the sound pressure level of different leakage diameters with a 45° jet direction and 2 m distance. It can be seen that: (1) The center of the spectrum is related to the leak diameters, but its amplitude is not greatly affected by the leak diameters. (2) Under the same pressure condition, the effect of leak diameter on the amplitude of the radiated noise SPL is very little. For example, the peak difference does not exceed 6 dB when the diameter varies from DN8 to DN25. (3) On the compact sound source, with the increase of the leakage hole diameter, the frequency bandwidth of the radiated noise becomes narrower, and the center of the spectrum moves toward the low-frequency direction. This phenomenon is similar to the situation when the pressure increases.

## 6. Identification of Time-Frequency Images

The convolutional neural network is a multilayer artificial neural network specially used to process two-dimensional input data [34,35]. In this study, the input of the CNN was a time-frequency matrix diagram of the reconstructed signal obtained by Hilbert transform, and the image was a color map with a size of 36 × 36 pixels. The time-frequency images under four different leakage conditions were input into the CNN to extract the features and combined with the radiated sound power level of the signal as the feature vector. A CNN model was then built to identify the patterns in the feature vectors. The Relu function was used after each convolutional layer, and each type of sample was labeled. Firstly, a convolutional layer with the kernel size of a × a was used to transform the input acoustic image from RGB space to feature space. After that, a Conv-Relu layer and a pooling layer were employed to extract the feature and reduce the dimension of the feature. This process was operated twice on CNN. At last, a fully connected layer and an output layer were used to provide the classification result. The categorical cross-entropy loss was used to calculate the output and label.

**Figure 17:**
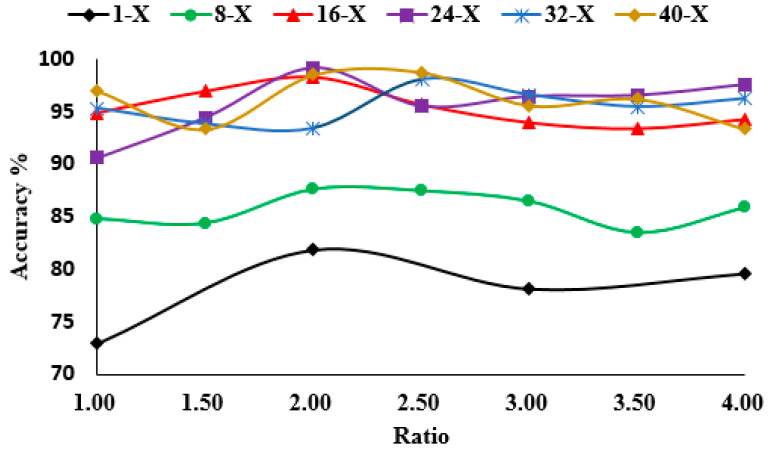
Validation accuracy at different ratios.

In the experiment, different combinations of structure collocation were investigated. In Figure 17, the model has a high accuracy rate only when the number of convolution kernels in the first layer is greater than or equal to 16, and the second layer has twice as many kernels as the first. As a result, considering the balance of the performance and efficiency, the architect of CNN was set as in Table 3.

**Figure 18:**
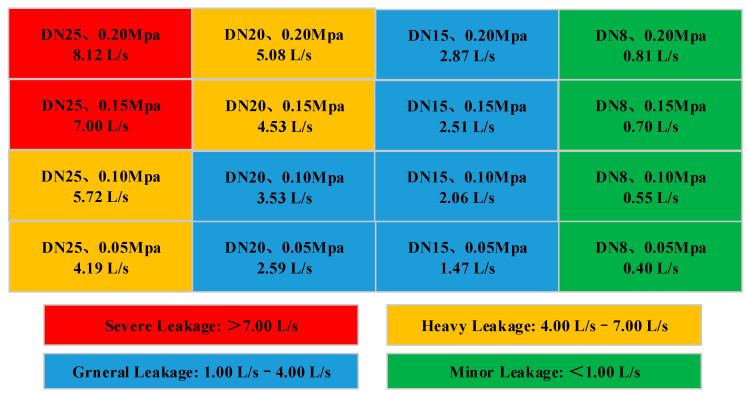
Leakage level groups.

In this study, we collected 3800 fault samples in a single leak and divided them into five groups (i.e., four leaked samples and one nonleaked sample) according to the degree of the leak, as shown in Figure 18. There are 760 samples in each group. Each type has 540 training samples, 100 validation samples and 120 testing samples.

**Figure 19:**
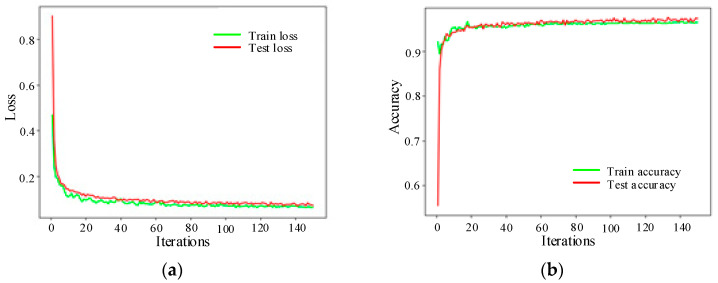
Leakage level identification. (**a**) Identification loss; (**b**) Identification accuracy

Among them, label1 = 1 of the data set corresponds to minor leakage, label2 = 2 corresponds to general leakage, label3 = 3 corresponds to heavy leakage, label4 = 4 corresponds to severe leakage, and label5 = 0 means no leakage. In Figure 19, after 150 iterations training, the prediction error is reduced from the original 0.88 to 0.09, and the prediction accuracy of the training set increases from 55% to 97%.

**Figure 20:**
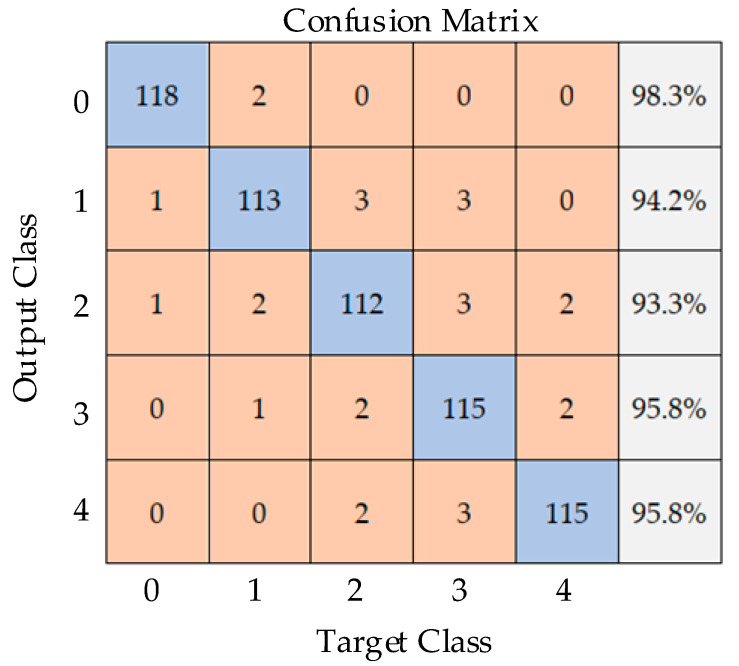
Confusion matrix.

Six-hundred samples from testing data are used to identify the leakage conditions, and the confusion matrix is shown in Figure 20. The number of successfully identified samples is 573, and the prediction accuracy is 95.5%. The feasibility of this method is proved.

## 7. Conclusions

In this paper, the mechanism of the underwater pipeline leakage radiation signal was studied experimentally, and the effect of leakage pressure and leakage aperture on leakage law is also analyzed. The main conclusions are as follows.

1. It is found that the radiation noise generated by the pipeline leakage is mid–high-frequency continuous spectrum noise. The increase of the pipeline pressure and the pipeline leakage diameter will cause the narrowing of the frequency bandwidth, and its center frequency moves toward the low-frequency direction. Under the same conditions, the pressure has a greater influence on the amplitude. For every 0.15 MPa increase in the pressure, the radiation sound pressure level increases by 6–7 dB.
2. According to the characteristics of the pipeline leakage acoustic signal, the signal is preprocessed. Through time-frequency analysis, a time-frequency image is obtained, which can accurately describe the characteristics of the signal. A simple two-layer network is built to train these images, and its recognition accuracy is validated at the degree of pipeline leakage.
3. The combination of CNN and the visualization of underwater acoustic signals in the time-frequency domain provides a novel insight for fault identification of underwater pipelines.
4. This method is mainly used to collect and analyze the jet noise generated by pipeline leakage, which is limited by the sensitivity of hydrophones in the working distance, and the acting depth is limited by transmission cable length. In the marine environment, if the leakage hole is too small, the generated noise may be drowned by the environmental noise, which induces the difficulty of identification. Therefore, it is necessary to select equipment according to local conditions, and the hydrophone should be not far away from the pipeline during testing.

One should note that in the present study the identification of the pipeline leakage location was not investigated yet. This is because in order to perform location identification we need to conduct different leakage location experiments; however, the pipeline with different leakage locations are currently not available. In the next step, the proposed method will be used to obtain acoustic images for different leakage distances, and a sufficient database will be used to train the CNN model for leakage localization.

## Figures and Tables

**Table 1:** Test equipment.

Name	Range	Accuracy
DHP8501 Digital hydrophone	20 Hz–20 kHz	±1.5 dB
ZB-0.10/8B Air compressor	0–0.8 MPa	–
WZB Pressure tank	0–108 L	–
Self-priming pump	0–30 m	–
Flowmeter	0–50 m^3^/h	±0.5%
Pressure gauge	0–1 MPa	±1%

**Table 2:** Leak hole diameter.

Name	DN25	DN20	DN15	DN8
Diameter (mm)	25	20	15	8

**Table 3:** Architect of CNN.

Layer	Layer Name	Layer Size
1	Input	875 × 656 × 3
2	Reshape	36 × 36 × 1
3	Convolution1	5 × 5 × 16
4	Max Pooling1	2 × 2 with stride 2
5	Convolution2	3 × 3 × 32
6	Max Pooling2	2 × 2 with stride 2
7	Fully connected layer	128
8	Softmax	–
9	Classify output layer	–
